# Evaluating pharmacological targeting of actin regulatory pathways during aging in *C. elegans*

**DOI:** 10.1007/s10522-026-10503-3

**Published:** 2026-09-02

**Authors:** Tiffany Wang, Athena Alcala, Daniella Berdan, Veronica Kuo, Gilberto Garcia, Ryo Higuchi-Sanabria, Maxim Averbukh

**Affiliations:** Leonard Davis School of Gerontology, University of Southern California, Los Angeles, CA 90089 USA

**Keywords:** Actin, Aging, Microscopy

## Abstract

Actin is a highly conserved cytoskeletal protein that regulates numerous cellular processes essential for tissue homeostasis and healthy aging. Although genetic studies have established important roles for actin and actin-binding proteins in longevity, pharmacological approaches offer complementary advantages for manipulating conserved cytoskeletal pathways across model systems. Here, we systematically evaluated the effects of small-molecule modulators targeting distinct actin regulatory pathways on lifespan, locomotor function, and tissue-specific actin organization in *Caenorhabditis elegans*. Animals were treated with compounds targeting the Arp2/3 complex (CK666), tropomyosin-dependent actin regulation (TR100), formins (SMIFH2), cofilin regulation (SZ-3), or actin stabilization (phalloidin). Among the compounds tested, only CK666 consistently produced a dose-dependent reduction in lifespan and exacerbated age-associated muscle actin disorganization, identifying Arp2/3-mediated actin branching as a critical regulator of cytoskeletal integrity during aging. CK666 also transiently disrupted hypodermal actin organization early in adulthood. In contrast, SMIFH2, TR100, phalloidin, and SZ-3 produced little or no detectable disruption of muscle actin organization, despite previous genetic studies demonstrating roles for several of these pathways in aging. Comparison with prior RNAi studies suggests that pharmacological perturbation can reproduce some aspects of actin dysfunction but may be limited by compound stability, drug delivery, or inefficient targeting of proteins in *C. elegans*. Together, these findings establish a framework for evaluating pharmacological modulation of actin during aging, identify CK666-mediated Arp2/3 inhibition as the most robust pharmacological perturbation under the conditions tested, and highlight important considerations for translating genetic discoveries into pharmacological strategies to target cytoskeletal function during aging.

**Supplementary Information:**

The online version contains supplementary material available at https://doi.org/10.1007/s10522-026-10503-3.

## Introduction

Actin is a dynamic and ubiquitous protein regulating a multitude of cellular processes, including cytoskeletal integrity, cell motility, proliferation, and facilitating intracellular transport (Amberg et al. 2012; Dominguez and Holmes 2011; Pollard 2016). The regulation of actin dynamics is essential for cellular function and longevity. Actin exists as globular actin (G-actin), the monomeric form, and filamentous actin (F-actin), the polymerized form, intricately maintaining a dynamic equilibrium between the two (Dominguez and Holmes 2011).

Actin dysregulation manifests in aging in multiple complex ways, as it serves numerous functions in various cells and tissues, including providing structural support to cells, intracellular cargo trafficking, and both intracellular and intercellular signaling. In vertebrate systems, aging phenotypes such as neuronal decline, muscle wasting, dysregulated proliferation, and immune dysfunction all involve pathological changes in actin-dependent processes (Averbukh, et al. 2026). For example, brains from patients with Alzheimer’s Disease (AD) may contain abnormal cofilin-rich actin rods, and actin dysfunction can drive defects in neuronal development and neuroplasticity (Bernstein et al. 2012). Proper actin function and organization are also important for synthesis and maintenance of dendritic spines and is strongly correlated with synaptic plasticity (Hotulainen and Hoogenraad 2010) and actin disorganization can result in neurological decline from decreased gray matter in aging. Sarcopenia, another age-related disease characterized by muscle wasting, is also highly correlated with actin and myosin fiber dysfunction. Actin and myosin serve as the main contractile proteins responsible for muscle cell movement and overall strength support (Teixeira et al. 2012), and age-associated loss of muscle function can be correlated with gene variability in the β-actin locus (Averbukh, et al. 2026). Actin also plays an important role in cell signaling, particularly within the immune system. Dynamic rearrangement of actin filaments is required for immune cells to alter their shape, migrate, and become activated, supporting coordinated responses to stimuli (Moulding et al. 2013). Finally, actin is essential for cell division as it contributes to cytoskeletal organization and forms the contractile ring during cytokinesis (López-Otín et al. 2013). With aging, regulation of cell proliferation can become disrupted, leading to increased mutation load, altered signaling pathways, and a higher risk of tumor development (López-Otín et al. 2013; Zhang et al. 2025).

The dynamic functions of actin are supported by the concerted effort of a diverse network of actin-binding proteins that regulate filament assembly, organization, and turnover. These regulators can be broadly categorized into proteins that control actin monomer arrangement, nucleate new filaments, promote elongation, stabilize existing structures, or drive filament disassembly (Dominguez and Holmes 2011). Among the primary actin nucleators, formin proteins generate long, unbranched filaments through processive elongation at the barbed end, while the Arp2/3 complex initiates branched filament networks that support complex cytoskeletal architectures (Pollard 2016; Chesarone and Goode 2009). As filaments mature, stabilizing proteins such as tropomyosin and troponin bind and stabilize filaments or regulate interactions with other cytoskeletal components. In contrast, actin turnover is promoted by cofilin and actin-depolymerizing factors, which sever filaments and enhance monomer recycling (Pollard 2016; Alaqel et al. 2022). Precise coordination across dozens of proteins is essential for maintaining cytoskeletal plasticity, and disruptions in actin regulation have been linked to cellular dysfunction, tissue degeneration, aging-related pathology, and disease (Amberg et al. 2012; Bamburg and Bernstein 2016; Gourlay and Ayscough 2005).

Despite extensive correlative evidence linking actin dysregulation to aging pathology, direct pathway-specific effects of actin changes in aging have only begun to be understood. For example, HSF-1 is a stress-responsive transcription factor that was first discovered to regulate the cytosolic heat shock response. Importantly, overexpression of *hsf-1* has been shown to extend lifespan through activation of cellular stress responses and improved actin stability (Baird et al. 2014). HSF-1 regulates *pat-10*, a gene encoding a cytoskeletal maintenance protein that, when overexpressed, can improve actin function to enhance tolerance to stress and promote longevity (Kuwahara, et al. 2008). Similarly, BET-1, a bromodomain protein, also transcriptionally alters actin-related gene expression (Garcia et al. 2023). *bet-1* overexpression in *C. elegans* results in improved actin maintenance with age, which promotes longevity (Garcia et al. 2023). Related studies in *Drosophila* examining brain aging under longevity-promoting conditions, such as dietary restriction and rapamycin treatment, have found a decrease in F-actin levels in the brain. In contrast, animals on high-protein diets, which are associated with shorter lifespans, exhibit increased F-actin levels beginning in middle to late age. Brains of young animals did not report any F-actin rods, supporting the impact of actin in aging pathology (Schmid et al. 2024).

Most recently, our lab utilized RNAi knockdown of genes encoding actin-binding proteins in *C. elegans* to determine the impact of actin dysfunction on aging. We found that targeted disruption of actin resulted in a general decrease in lifespan and exacerbation of the biological hallmarks of aging, with some nuanced tissue-specific effects, highlighting the role of actin as a central modulator of aging phenotypes and healthspan (Averbukh, et al. 2026).

However, RNAi approaches have several limitations, including inefficient delivery in certain tissues, incomplete or transient silencing, off-target effects, and compensation by isoforms or proteins with redundant functions (Kamath and Ahringer 2003). In addition, precise stabilization and destabilization of actin filaments is difficult to manipulate in vivo through genomic approaches, particularly in human systems or animal models where genetic tools are not readily available. As a complementary approach to RNAi, chemical strategies offer rapid and tunable control over actin dynamics. Small molecules targeting actin polymerization, depolymerization, or actin-binding proteins can be applied acutely and titrated to modulate filament stability across a range of conditions, facilitating dose-dependent analyses of cytoskeletal function. Moreover, chemical approaches can circumvent issues of genetic redundancy by directly targeting conserved biochemical activities, and they are readily deployable across diverse model systems.

Here, we tested a suite of small molecules targeting distinct regulators of actin assembly, stability, and turnover to determine their effects on longevity and cytoskeletal organization in *C. elegans*. Specifically, we utilized CK666 to inhibit Arp2/3-mediated actin branching (Hetrick et al. 2013); TR100, originally identified as an inhibitor of the cancer-associated tropomyosin isoform Tpm3.1, to target tropomyosin-dependent actin regulation (Rynkiewicz et al. 2017; Stehn et al. 2013); SMIFH2 to target formin-mediated actin nucleation and elongation (Isogai et al. 2015); and SZ-3 to target cofilin regulation (Alaqel et al. 2022). In contrast, phalloidin was used to stabilize filamentous actin (Bubb et al. 1994; Pospich et al. 2020; Holzinger 2009). Together, these compounds enabled us to systematically evaluate the feasibility and consequences of pharmacologically targeting distinct mechanisms of actin regulation in *C. elegans*.

## Methods

### *C. elegans* maintenance

This study used the Bristol N2 wild-type strains obtained from the *Caenorhabditis* Genetics Center (CGC). Animals were maintained on nematode growth medium (NGM) plates at 15 °C and transferred to fresh plates on a weekly basis. The plates were seeded with *Escherichia coli* OP50 (B strain) cultured in Luria–Bertani (LB) medium for 24–48 h at room temperature. To prevent genetic drift, >20 individual worms were moved onto fresh OP50 plates every week for no more than 25–30 generations. Experimental strains were synchronized using a standard bleaching protocol to remove OP50 bacteria and subsequently cultured at 20 °C on *E. coli* K strain (Ht115) bacteria (Castro Torres et al. 2022). For all experiments, Ht115 carrying an empty vector (EV) pL4440 RNAi construct was used. All experiments were performed on Ht115 bacteria so that they can be easily compared to previous studies using RNAi treatment. All strains used in this study are listed in Table S1.

### Plates

Plates were prepared using BD Difco granulated agar (VWR, 90,000–782), as the agar source can influence substrate stiffness and *C. elegans* physiology (Oorloff et al. 2024). Agar components were mixed, sterilized by autoclaving, and cooled to approximately 60 °C before supplementation with salts, antibiotics, IPTG, and drug solutions. Drugs were reconstituted from stock solutions and diluted in DMSO to achieve the desired final concentrations prior to incorporation into the molten agar. Drugs and/or DMSO were added to molten agar once cooled to approximately 47 °C. The agar was then poured into plates, allowed to solidify and dry, and stored at 4 °C until use. All drug plates were prepared fresh and used immediately for experimentation. All drugs used in this study are listed in Table S2.

#### Standard NGM plates for maintenance

Bacto-Agar (Difco) 2% w/v, Bacto-Peptone 0.25% w/v, NaCl 0.3% w/v, 1 mM CaCl_2_, 5 µg/mL cholesterol, 0.625 mM KH_2_PO_4_ (pH 6.0), 1 mM MgSO₄.

#### RNAi plates for experiments

Bacto-Agar (Difco) 2% w/v, Bacto-Peptone 0.25% w/v, NaCl 0.3% w/v, 1 mM CaCl_2_, 5 µg/mL cholesterol, 0.625 mM KH₂PO₄ (pH 6.0), 1 mM MgSO_4_, 100 µg/mL carbenicillin, 1 mM IPTG.

#### DMSO plates for experiments

Bacto-Agar (Difco) 2% w/v, Bacto-Peptone 0.25% w/v, NaCl 0.3% w/v, 1 mM CaCl_2_, 5 µg/mL cholesterol, 0.625 mM KH₂PO₄ (pH 6.0), 1 mM MgSO_4_, 100 µg/mL carbenicillin, 1 mM IPTG. Dimethyl sulfoxide (DMSO) was used as a solvent control and added in volumes equivalent to those used for drug-containing plates prior to plate pouring.

#### CK666 drug plates for experiments

Bacto-Agar (Difco) 2% w/v, Bacto-Peptone 0.25% w/v, NaCl 0.3% w/v, 1 mM CaCl_2_, 5 µg/mL cholesterol, 0.625 mM KH₂PO₄ (pH 6.0), 1 mM MgSO_4_, 100 µg/mL carbenicillin, 1 mM IPTG. CK666 was reconstituted to a 30 mM stock solution, diluted, and mixed into molten agar to final concentrations of 0.5, 1, 2.5, 5, 50, and 100 µM prior to plate pouring.

#### SMIFH2 drug plates for experiments

Bacto-Agar (Difco) 2% w/v, Bacto-Peptone 0.25% w/v, NaCl 0.3% w/v, 1 mM CaCl₂, 5 µg/mL cholesterol, 0.625 mM KH₂PO₄ (pH 6.0), 1 mM MgSO₄, 100 µg/mL carbenicillin, 1 mM IPTG. SMIFH2 was reconstituted to a 30 mM stock solution and mixed into molten agar to final concentrations of 1, 2.5, 5, and 10 µM prior to plate pouring.

#### SZ-3 drug plates for experiments

Bacto-Agar (Difco) 2% w/v, Bacto-Peptone 0.25% w/v, NaCl 0.3% w/v, 1 mM CaCl₂, 5 µg/mL cholesterol, 0.625 mM KH₂PO₄ (pH 6.0), 1 mM MgSO₄, 100 µg/mL carbenicillin, 1 mM IPTG. SZ-3 was reconstituted to a 30 mM stock solution and mixed into molten agar to final concentrations of 1, 2.5, 5, and 10 µM prior to plate pouring.

#### TR100 drug plates for experiments

Bacto-Agar (Difco) 2% w/v, Bacto-Peptone 0.25% w/v, NaCl 0.3% w/v, 1 mM CaCl₂, 5 µg/mL cholesterol, 0.625 mM KH₂PO₄ (pH 6.0), 1 mM MgSO₄, 100 µg/mL carbenicillin, 1 mM IPTG. TR100 was reconstituted to a 30 mM stock solution and mixed into molten agar to final concentrations of 1, 2.5, 5, and 10 µM prior to plate pouring.

#### Phalloidin drug plates for experiments

Bacto-Agar (Difco) 2% w/v, Bacto-Peptone 0.25% w/v, NaCl 0.3% w/v, 1 mM CaCl_2_, 5 µg/mL cholesterol, 0.625 mM KH_2_PO_4_ (pH 6.0), 1 mM MgSO_4_, 100 µg/mL carbenicillin, 1 mM IPTG. Phalloidin was reconstituted to a 30 mM stock solution and mixed into molten agar to final concentrations of 1, 2.5, 5, and 10 µM prior to plate pouring.

### Bleaching

A standard bleaching protocol was used to synchronize *C. elegans* populations. Gravid adults were collected in M9 buffer (22 mM KH₂PO₄, 42.3 mM Na₂HPO₄, 85.6 mM NaCl, 1 mM MgSO_4_) and exposed to a freshly prepared bleaching solution containing 1.8% sodium hypochlorite and 0.375 M NaOH. Animals were shaken in the tubes until adult carcasses were dissolved and only eggs remained. Eggs were pelleted by centrifugation at 1,100 × g and washed four times with M9 buffer to remove residual bleach. After the final wash, eggs were resuspended in fresh M9 buffer and incubated overnight (up to 16 h) at room temperature (20 °C) on a shaker until synchronization at the L1 larval stage. Following synchronization, L1 larvae were plated onto respective plates.

### Lifespan assays

Animals were grown on treatment plates from the L1 stage, with approximately 120 worms distributed evenly across multiple plates (either six plates of 20 animals or four plates of 30 animals per condition). Animals were maintained at 20 °C and scored for survival beginning on day 5 of adulthood and every other day thereafter until all animals had been scored. Worms were scored as dead when they failed to respond to gentle prodding with a platinum wire. Care was taken to distinguish dead animals from aged worms exhibiting slowed movement. Animals were scored as censored if they displayed intestinal herniation, crawling up the sides of the petri dish, or other non-aging related deaths. Processed data were imported into GraphPad Prism to generate lifespan curves and perform statistical analyses (Log-Rank testing). All lifespan assays were performed using three independent biological replicates, and representative survival curves are shown in figures, with all lifespan data available in Table S3.

### Widefield imaging

For microscopy, animals were transferred directly onto glass microscope slides and anesthetized in M9 buffer containing 0.1 M sodium azide as previously described (Kim et al. 2025). Widefield fluorescence imaging was performed using a Leica Thunder Imager equipped with a 63×/1.4 Plan ApoChromat oil-immersion objective, dsRed and GFP filter sets, a Leica DFC9000 GT camera, and a Leica LED5 illumination system, controlled using LAS X software. To ensure standardization across samples, imaging was restricted to the region between the anterior head and the midpoint near the gonad.

While imaging procedures were largely consistent between muscle and hypodermal tissues, differences in tissue structure required distinct handling and evaluation criteria. Actin organization in the hypodermis exhibited diffuse, star-like patterns, in contrast to the highly ordered, striated architecture observed in muscle tissue. These tissue-specific differences were taken into account during imaging and subsequent quantification.

### Muscle quantification

Muscle actin quantification was completed through manual count of the number of muscle cells with defective actin-rich striations compared to the total number of muscle cells. Muscle actin organization was considered disrupted when observable abnormalities were present, including fraying, breaks, uneven striations, or missing striations. For each image, the percentage of muscle cells with disrupted actin striations relative to the total number of muscle cells per image was calculated. Each single data point is the average across all animals in a single biological replicate. Statistical analyses were performed using GraphPad Prism, applying a non-parametric Mann–Whitney U test.

### Hypodermal quantification

Hypodermal actin organization was quantified using Fiji software by calculating the proportion of fluorescent signal relative to the total image area as previously described (Averbukh, et al. 2026). Percent changes were determined by comparing each experimental condition to the corresponding control group. Quantified data were subsequently analyzed and plotted using GraphPad Prism, and statistical significance was assessed using analysis of variance (ANOVA).

### Thrash

To quantify locomotor behavior of animals, liquid thrashing assays were performed on days 1, 5, and 9 (D1, D5, D9) of adulthood. Adult animals on experimental plates were flooded with 100 µL of M9 solution, and 30-s videos were recorded using a Leica K5 camera mounted on a Leica M205FCA stereomicroscope. Thrashing was measured manually by counting the number of single thrashes during a 10-s period. A complete thrash was defined as a bend in which the adult animal’s body bent more than 50% in opposite directions. Data are represented from three biological replicates and plotted using GraphPad Prism 7 as a dot plot where each dot represents a single animal and lines represent median and interquartile range. Statistical analysis was performed using a nonparametric Mann–Whitney test.

## Results

### Pharmacological targeting of actin regulatory proteins produces differential effects on lifespan

Standard lifespan assays were performed in *C. elegans* to evaluate the impact of pharmacological perturbation of actin on organismal longevity. In previous studies, we found that a 1–5 µM concentration range of Latrunculin A and Jasplakinolide were sufficient to have dose-dependent effects on lifespans (Averbukh, et al. 2026). Therefore, we started with a similar concentration range for TR100, SZ-3, phalloidin, CK666, and SMIFH2, as well as additional higher concentrations for compounds that were not cost-prohibitive and did not have a lifespan effect at the 1–5 µM range.

**Fig. 1:**
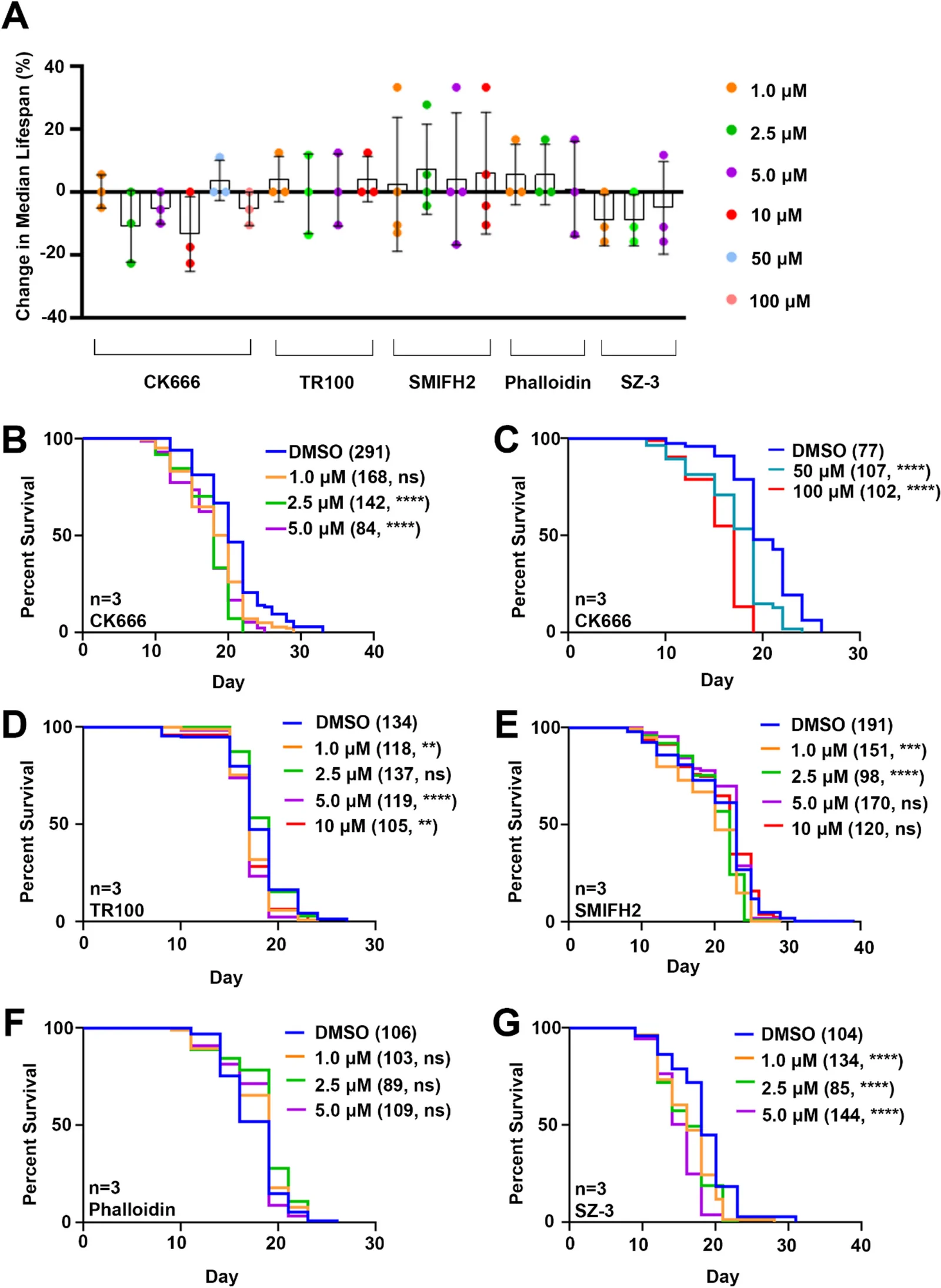
Pharmacological targeting of actin regulatory proteins produces differential effects on lifespan in *C. elegans*. **A** Average median lifespan change (% MLS change) in wild-type animals following exposure to the small molecules CK666, TR100, SMIFH2, Phalloidin, and SZ-3, compared with the DMSO control. Concentrations ranged from 1–100 µM: DMSO (blue), 1.0 µM (orange), 2.5 µM (green), 5.0 µM (purple), 10 µM (red), 50 µM (light blue), and 100 µM (pink). Error bars indicate the standard deviation (s.d.). **B-G** Lifespan curves of animals exposed from L1 to **B** low concentrations of CK666 (10 µM lifespan is in Fig. S1), **C** high concentrations of CK666, **D** TR100, **E** SMIFH2, **F** Phalloidin, and **G** SZ-3 while grown on empty vector (EV) from L1. ns = p > 0.05, ** = p < 0.01, *** = P < 0.001, **** = p < 0.0001 using Log-Rank Testing

Lifespan assays of actin-targeting compounds showed variable impacts on lifespan. Treatment with the Arp2/3 inhibitor CK666 resulted in a dose-dependent decrease in lifespan, where concentrations lower than 5.0 µM had only minimal effects on lifespan and concentrations higher than 5 µM showed a significant lifespan reduction (Fig. 1A-C, Fig. S1). However, all other drugs had either only minimal effects or no significant effects on lifespan. Treatment with TR100 (Fig. 1A, D), formin inhibitor SMIFH2 (Fig. 1A, E), and phalloidin (Fig. 1A, F), which competes with cofilin binding and thus can stabilize actin filaments, had no consistently significant impact on lifespan. The cofilin inhibitor SZ-3 did not have a major impact on lifespan, although most replicates showed a modest but statistically significant reduction in lifespan (Fig. 1A, G). Overall, with the exception of CK666 and SZ-3, most pharmacological perturbations of actin regulation did not have major impacts on longevity, unlike previous studies that utilized genetic methods to target actin (Averbukh, et al. 2026).

**Fig. 2:**
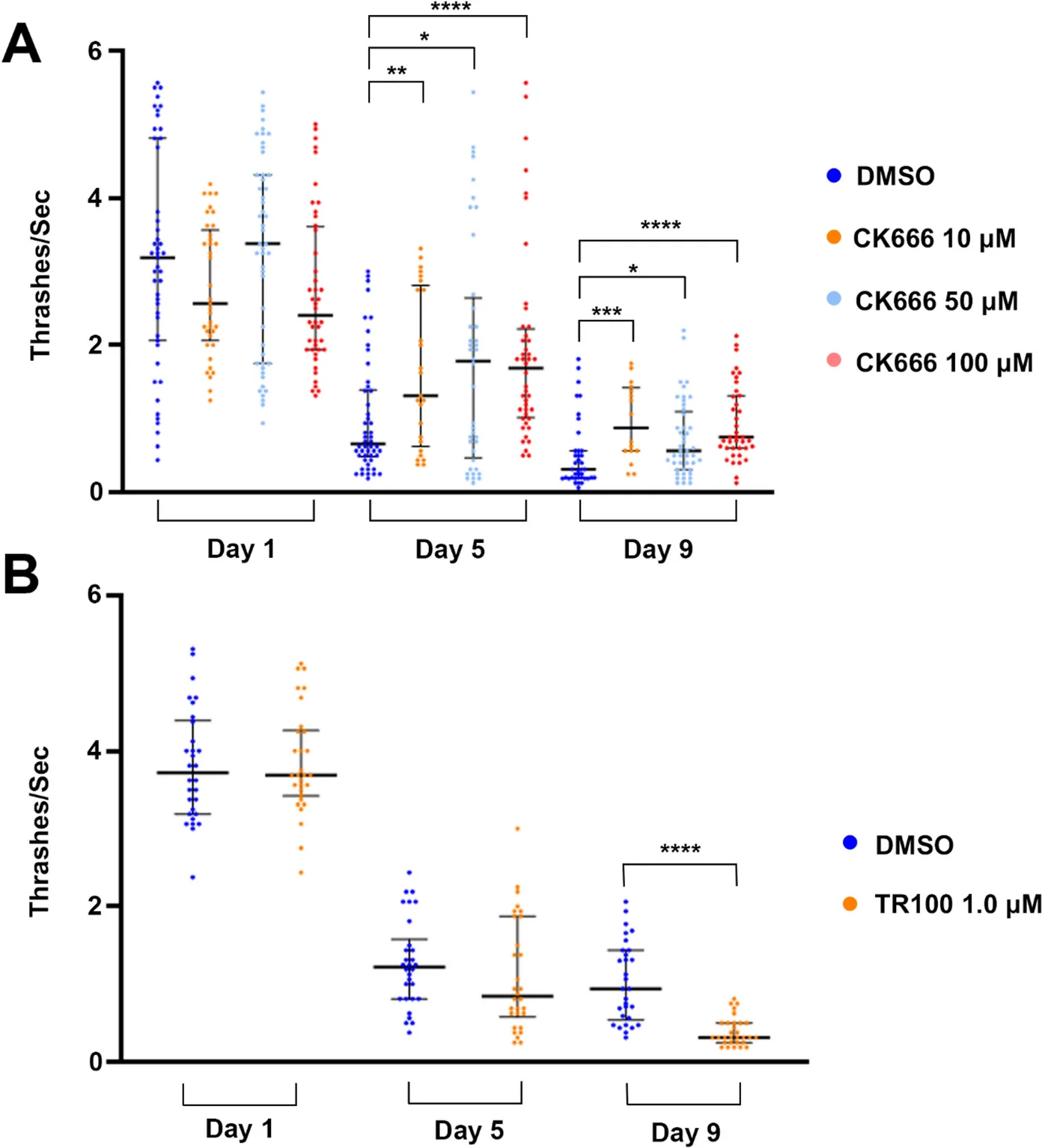
CK666 and TR100 differentially alter age-dependent locomotor function. (A, B) Thrashing assays were performed on N2 animals grown on empty vector (EV) from L1. Animals were treated with DMSO control (blue) and 10 µM (orange), 50 µM (light blue), or 100 µM (pink) CK666 (**A**) or 1.0 µM (orange) TR100 (**B**). Thrashing was measured on days 1, 5, and 9 of adulthood. Recordings were performed in M9 buffer, and thrashing was manually scored over a 10-s recording and normalized to thrashes per second. Data are representative of three independent trials. N = 30–33 worms per condition. Each dot represents a single animal, and lines indicate the median and interquartile range. * = p < 0.05, ** = p < 0.01, *** = P < 0.001, **** = p < 0.0001 using non-parametric Mann–Whitney test

To determine whether these lifespan effects were accompanied by functional changes in organismal physiology, we next assessed locomotor activity. Because actin dynamics are critical for muscle contraction and whole-animal movement, we focused on CK666, the compound that produced the most pronounced effects on lifespan, and TR100, which impacts tropomyosin, an important component in regulating actin-myosin interactions for muscle contractions (Bonello et al. 2016). We performed liquid thrashing assays to evaluate the impact of pharmacological perturbations of actin on motility. Interestingly, we found that CK666 resulted in a mild but statistically significant increase in motility on days 5 and 9 (Fig. 2a). However, TR100 resulted in an expected reduction in motility on day 9 of adulthood (Fig. 2b). These data suggest that changes in motility are not directly correlated with a lifespan outcome when perturbing actin using pharmacological agents.

### Pharmacological targeting of actin regulators reveals selective effects on muscle and hypodermal actin architecture

The limited impact of pharmacological perturbation on lifespan may be due to insufficient drug delivery, potentially due to the impermeability and negative charge of the *C. elegans* cuticle (Gonzalez-Moragas et al. 2015; Artal-Sanz et al. 2006). Therefore, to evaluate whether these compounds successfully altered actin, we performed in vivo imaging of actin organization to assess structural integrity across aging. Here, we used transgenic lines expressing LifeAct::mRuby, which allows robust tissue-specific imaging of actin structure and organization (Higuchi-Sanabria R, et al. 2018). Actin in *C. elegans* muscle cells displays a highly organized, striated structure that runs in parallel along myosin filaments. During aging, this organization becomes progressively disrupted, manifesting as fragmentation, loss of parallel alignment, and increased waviness of actin-rich striations.

**Fig. 3:**
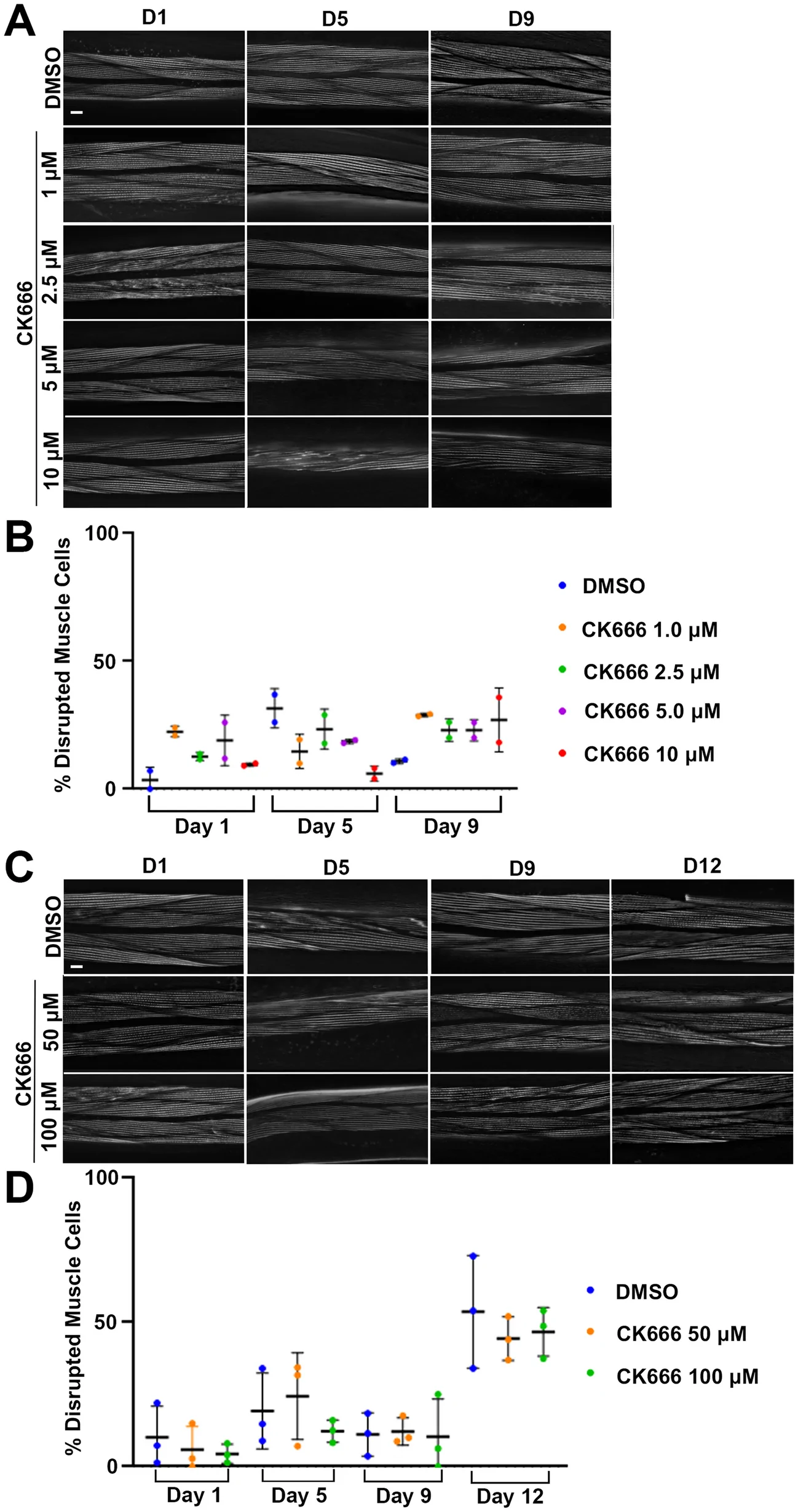
CK666 exacerbates age-associated muscle actin disorganization. (**A**) Representative fluorescence images of adult animals expressing LifeAct::mRuby from a muscle-specific (myo-3p) promoter, acquired on days 1, 5, and 9 of adulthood with DMSO control (blue), or 1.0 µM (orange), 2.5 µM (green), 5.0 µM (purple), or 10.0 µM (red) CK666. Data are representative of two biological replicates. Note: only two replicates were performed for low concentrations of CK666 as they were highly consistent with high concentration data, where three independent replicates were performed. (**B**) Quantification of (**A**) muscle actin striation disruption. (**C**) Representative fluorescence images of adult animals expressing LifeAct::mRuby from a muscle-specific (myo-3p) promoter, acquired on days 1, 5, 9, and 12 of adulthood with DMSO control (blue) or 50 µM (light blue) or 100 µM (pink) CK666. Data are representative of three biological replicates. (**D**) Quantification of (**C**) muscle actin striation disruption

Consistent with our lifespan data, CK666 treatment exacerbated age-associated muscle actin disorganization compared with DMSO-treated controls, with the effect increasing up to 10 μM. While control animals exhibited the expected age-related decline in actin organization, animals treated with 10 μM CK666 displayed markedly more severe fragmentation and disorganization of muscle actin striations in old age, with no further worsening observed at higher concentrations (Fig. 3A, C). Despite these pronounced morphological differences, the percentage of muscle cells classified as containing disrupted actin striations was not significantly different between CK666-treated and control animals (Fig. 3B, D). These findings suggest that CK666 does not increase the frequency of actin disruption across muscle cells but instead exacerbates the severity of actin striation disorganization within affected cells.

**Fig. 4:**
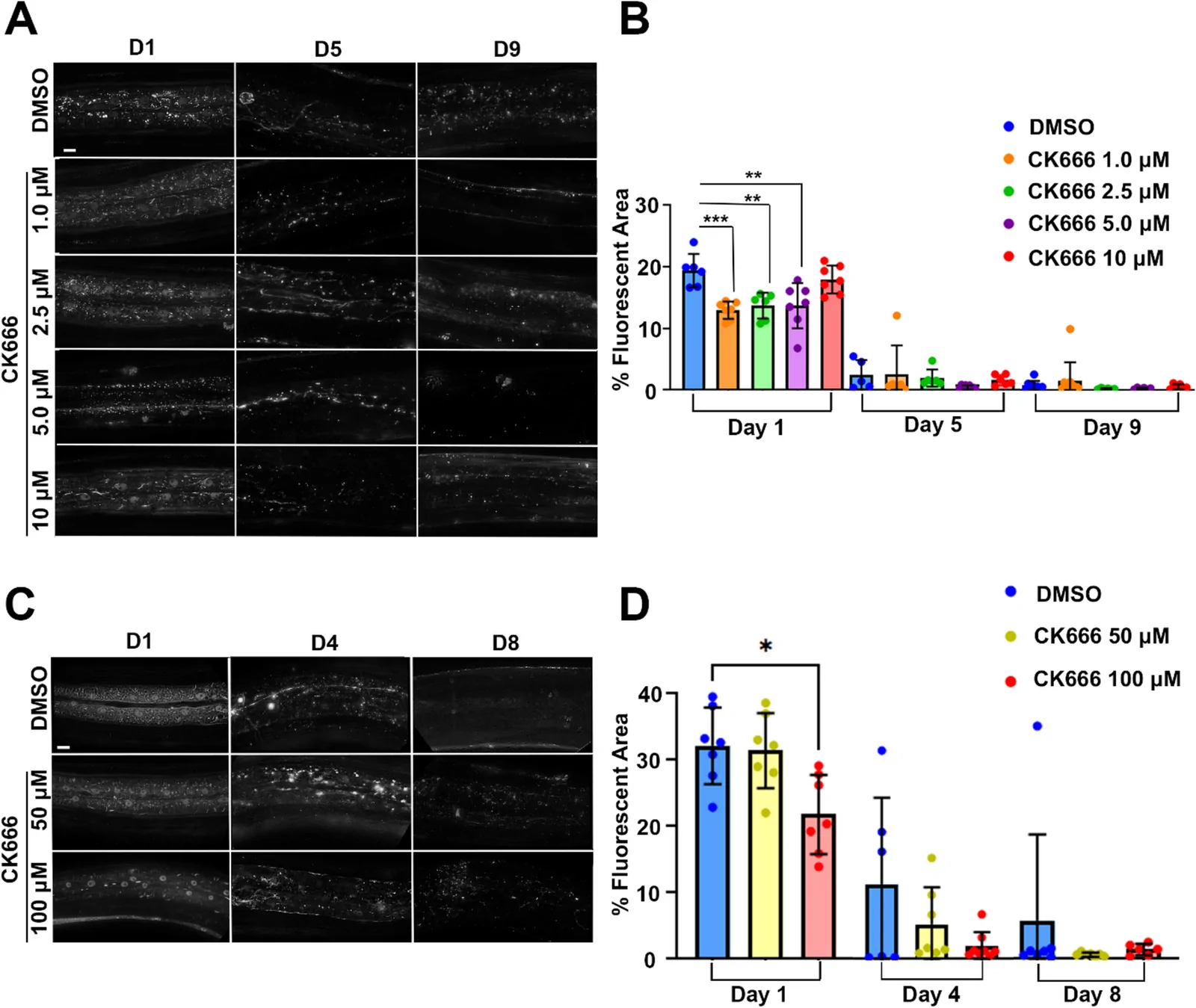
CK666 transiently disrupts hypodermal actin organization during early adulthood. **A** Representative fluorescence images of adult animals expressing LifeAct::mRuby from a hypodermis-specific (*col-19p*) promoter, acquired on days 1, 5, and 9 of adulthood, and quantification of **B** hypodermal actin organization following treatment with low concentrations of CK666. Concentrations were DMSO control (blue), 1.0 µM (orange), 2.5 µM (green), 5.0 µM (purple), and 10.0 µM (red). **C** Representative fluorescence images of adult animals expressing LifeAct::mRuby from a hypodermis-specific (*col-19p*) promoter, acquired on days 1, 4, and 8 of adulthood, and quantification of **D** hypodermal actin organization following treatment with high concentrations of CK666. Concentrations were DMSO control (blue), 50 µM (light blue), and 100 µM (pink)

To determine whether the effects of Arp2/3 inhibition were restricted to muscle, we next examined actin organization in the hypodermis. CK666 treatment resulted in a significant reduction in measurable hypodermal actin structures at day 1 of adulthood; however, this effect was not maintained at mid or late adulthood (Fig. 4). These findings suggest that CK666 induces a transient disruption of hypodermal actin early in adulthood but not sustained alterations in hypodermal actin organization over the course of aging.

**Fig. 5:**
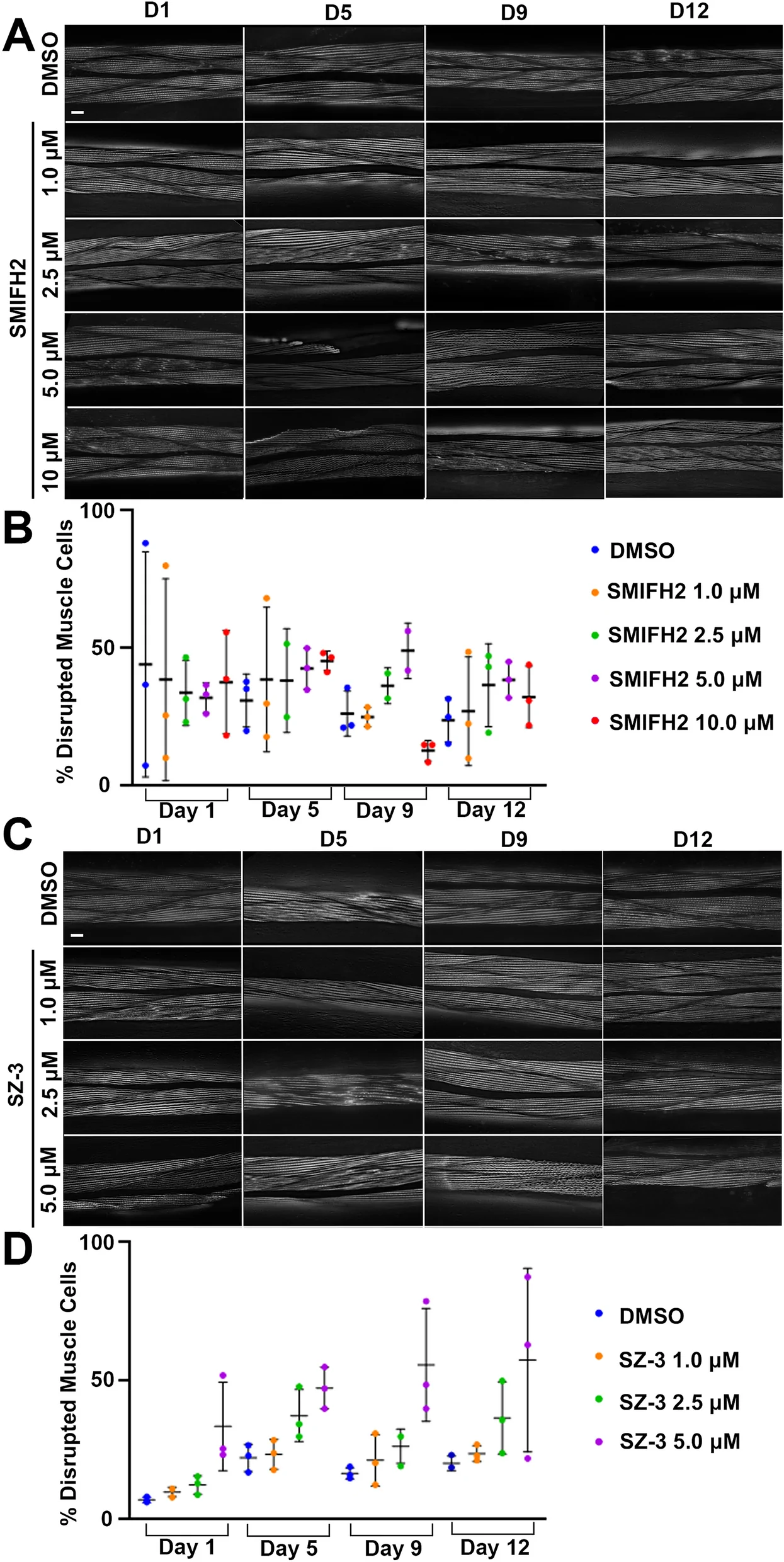
SMIFH2 and SZ-3 do not measurably alter muscle actin organization during aging. **A** Representative fluorescence images of adult animals expressing LifeAct::mRuby from a muscle-specific (*myo-3p*) promoter, acquired on days 1, 5, 9, and 12 of adulthood, and quantification of **B** muscle actin striation disruption following treatment with SMIFH2. Concentrations were DMSO control (blue), 1.0 µM (orange), 2.5 µM (green), 5.0 µM (purple), and 10.0 µM (red). **C** Representative fluorescence images of adult animals expressing LifeAct::mRuby from a muscle-specific (*myo-3p*) promoter, acquired on days 1, 5, 9, and 12 of adulthood, and quantification of **D** muscle actin striation disruption following treatment with SZ-3. Concentrations were DMSO control (blue), 1.0 µM (orange), 2.5 µM (green), and 5.0 µM (purple)

**Fig. 6:**
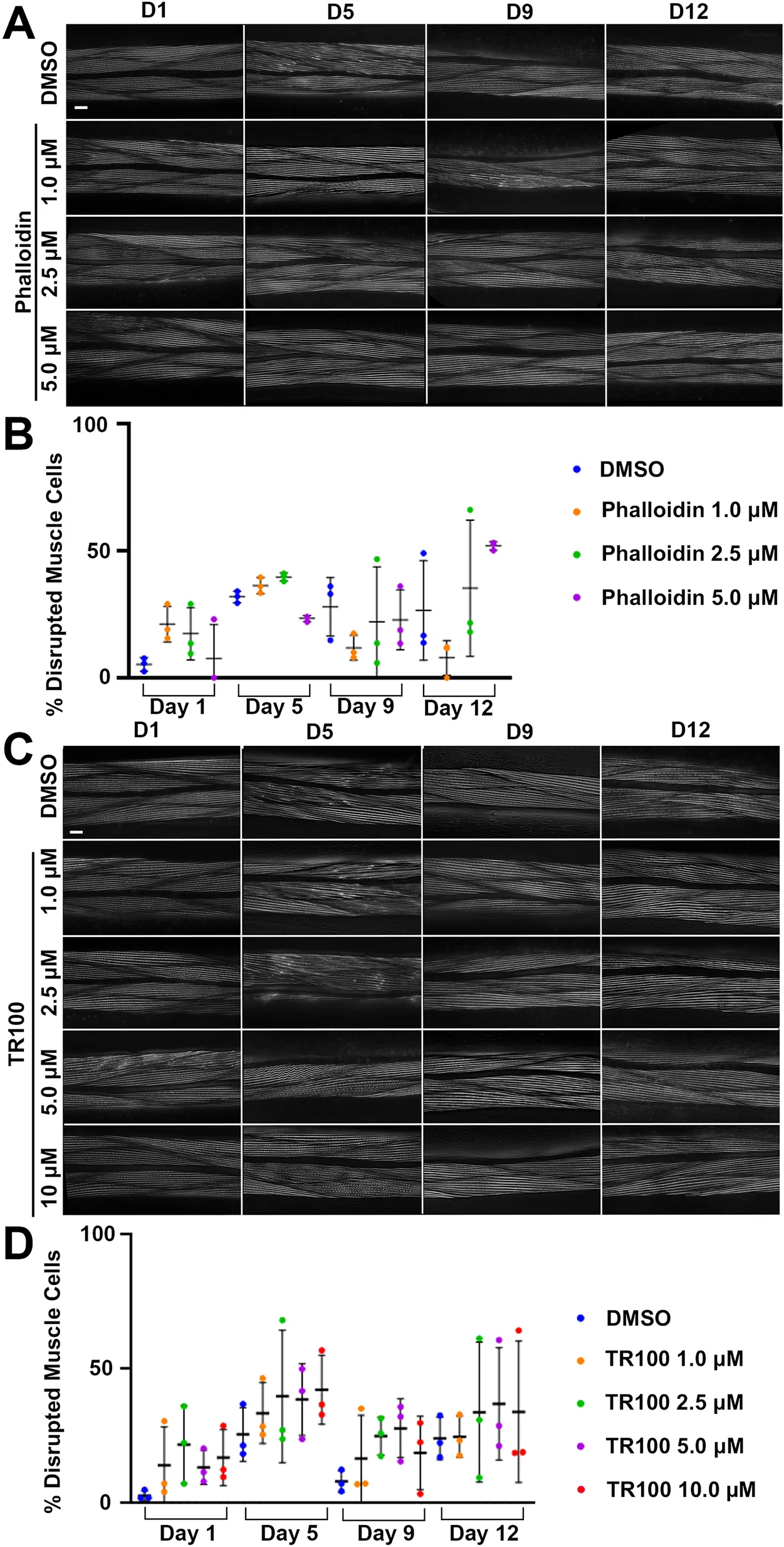
TR100 and phalloidin do not measurably alter muscle actin organization during aging. **A** Representative fluorescence images of adult animals expressing LifeAct::mRuby from a muscle-specific (*myo-3p*) promoter, acquired on days 1, 5, 9, and 12 of adulthood, and quantification of **B** muscle actin striation disruption following treatment with Phalloidin. Concentrations were DMSO control (blue), 1.0 µM (orange), 2.5 µM (green), and 5.0 µM (purple). **C** Representative fluorescence images of adult animals expressing LifeAct::mRuby from a muscle-specific (*myo-3p*) promoter, acquired on days 1, 5, 9, and 12 of adulthood, and quantification of **D** muscle actin striation disruption following treatment with TR100. Concentrations were DMSO control (blue), 1.0 µM (orange), 2.5 µM (green), 5.0 µM (purple), and 10.0 µM (red)

In contrast to CK666, treatment with SMIFH2 (Fig. 5A-B), SZ-3 (Fig. 5C-D), phalloidin (Fig. 6A-B), or TR100 (Fig. 6C-D), did not produce detectable changes in muscle actin organization at any age examined. These findings contrast with previous studies demonstrating robust muscle actin disruption following genetic inhibition of tropomyosin (*lev-11* RNAi) or cofilin (*unc-60* RNAi), or pharmacological actin stabilization using jasplakinolide (Averbukh, et al. 2026). Together, these results suggest that, under our experimental conditions, these compounds do not measurably perturb muscle actin organization.

**Fig. 7:**
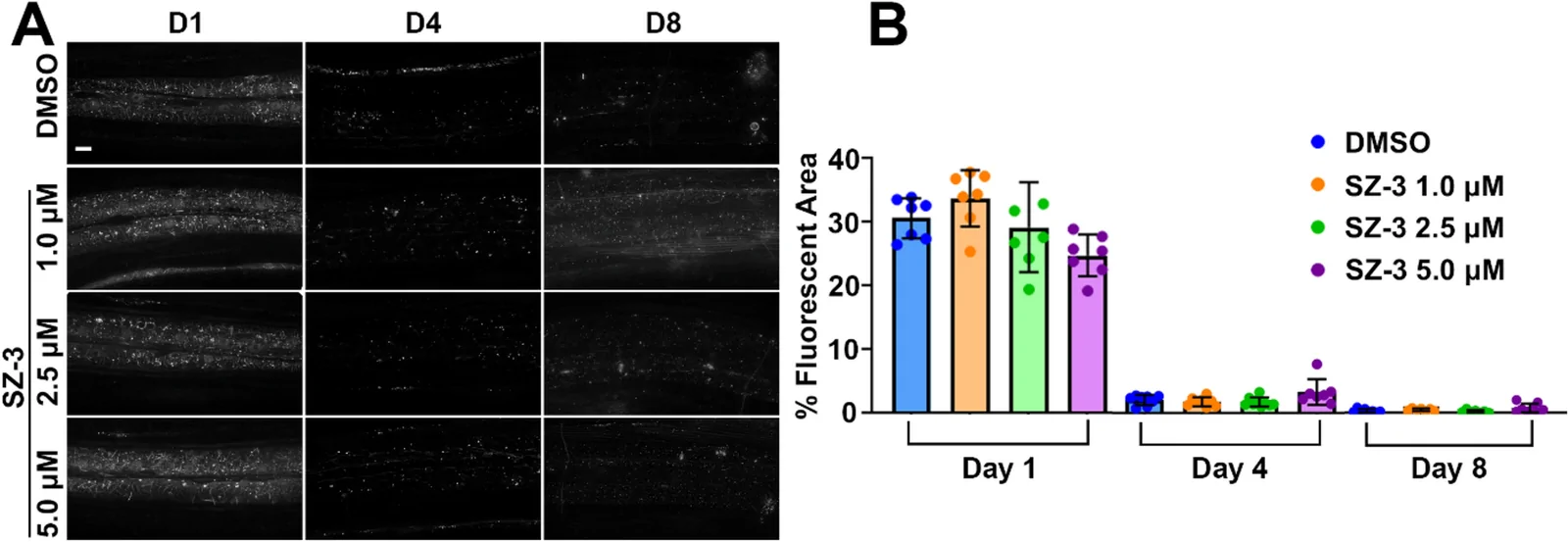
SZ-3 does not alter hypodermal actin organization. **A** Representative fluorescence images of adult animals expressing LifeAct::mRuby from a hypodermis-specific (*col-19p*) promoter, acquired on days 1, 4, and 8 of adulthood, and quantification of **B** hypodermal actin organization following treatment with SZ-3. Concentrations were DMSO control (blue), 1.0 µM (orange), 2.5 µM (green), and 5.0 µM (purple)

Because SZ-3 significantly shortened lifespan despite having no detectable effect on muscle actin, we next asked whether its activity might be restricted to other tissues. We therefore examined actin organization in the hypodermis. However, SZ-3 also failed to produce detectable changes in hypodermal actin organization at any age (Fig. 7). These findings indicate that the lifespan effects of SZ-3 are not accompanied by detectable alterations in either muscle or hypodermal actin organization, raising the possibility that its effects may occur through mechanisms distinct from overt actin disorganization.

## Discussion

In this study, we evaluated whether pharmacological perturbation of actin regulatory pathways could serve as an alternative to genetic approaches for investigating the role of the cytoskeleton in aging. By targeting distinct actin-binding proteins with small molecules, we sought to determine whether chronic chemical perturbation could recapitulate the lifespan and cytoskeletal phenotypes previously observed using RNAi-mediated knockdown of actin regulators. Overall, treatment with the Arp2/3 inhibitor CK666 consistently shortened lifespan and exacerbated age-associated muscle actin disorganization, whereas compounds targeting other actin regulatory mechanisms produced limited or no detectable effects on actin organization under our experimental conditions.

Comparison with our previous genetic studies revealed both important similarities and notable differences (Averbukh, et al. 2026). Consistent with RNAi-mediated knockdown of the Arp2/3 complex subunit *arx-2*, CK666 treatment shortened lifespan and increased muscle actin disorganization, supporting a conserved role for Arp2/3-mediated actin nucleation and branching in maintaining cytoskeletal integrity during aging. In contrast, treatment with the tropomyosin targeting compound TR100 did not reproduce the lifespan or muscle actin phenotypes observed following *lev-11* RNAi, despite targeting the same pathway. However, TR100 has been shown to selectively disrupt actin filaments containing the tropomyosin isoform Tm5NM1 (Tpm3.1), while sparing filaments containing the αTmfast isoform (Stehn et al. 2013). Protein sequence alignment against the *C. elegans* proteome using NCBI BLAST identified LEV-11 isoforms C/E as having the highest similarity to Tm5NM1, whereas LEV-11 isoforms A/B/D/F showed the highest similarity to αTmfast. Thus, differences in LEV-11 isoform expression, together with the isoform-selective activity of TR100, may contribute to the limited effects observed following TR100 treatment.

Notably, however, TR100 treatment reduced locomotor activity at day 9 of adulthood, indicating that the compound was not entirely without physiological effects under our experimental conditions. Given the role of tropomyosin in regulating actin–myosin interactions during muscle contraction, this phenotype could be consistent with a modest effect on muscle function that was insufficient to produce detectable changes in actin organization or longevity. However, because target engagement was not directly assessed, we cannot conclude whether the reduction in motility reflects inhibition of LEV-11 or another effect of TR100. Similarly, treatment with the cofilin-targeting compound SZ-3 resulted in only a modest reduction in lifespan without detectable disruption of muscle or hypodermal actin organization, differing from the stronger phenotypes observed following *unc-60* RNAi. Together, these findings demonstrate that CK666 treatment recapitulates key lifespan and muscle actin phenotypes observed following genetic disruption of Arp2/3, whereas compounds targeting several other actin regulators failed to fully reproduce the corresponding genetic phenotypes under our experimental conditions.

The limited effects observed for several compounds also contrast with studies performed in other model organisms. For example, manipulation of formin-dependent actin remodeling has been shown to improve neuronal health and maintain autophagy during aging in *Drosophila*, suggesting that regulation of actin dynamics can exert tissue-specific effects on organismal aging (Schmid et al. 2024). In contrast, SMIFH2 treatment did not measurably alter lifespan or muscle actin organization in *C. elegans*. These differences may reflect biological differences between tissues or organisms, but they also highlight an important limitation of whole-animal pharmacological treatment. Small molecules are delivered systemically and cannot readily distinguish between tissues, whereas aging phenotypes resulting from actin dysregulation may depend on perturbation within specific cell types.

Several technical factors may also explain why some pharmacological perturbations failed to reproduce genetic phenotypes. Drug stability represents one important consideration, particularly in long-term lifespan assays that require compounds to remain active for several weeks. Although fresh plates were prepared for every experiment and care was taken to minimize variability during plate preparation, factors including temperature, light exposure, and compound stability may influence effective drug concentrations over time. In addition, drug uptake remains a major challenge in *C. elegans*. The impermeable cuticle limits penetration of many compounds, potentially reducing intracellular concentrations below those required for effective target inhibition. This limitation may help explain why some compounds produced minimal phenotypic effects. For some compounds, the lack of an observable phenotype may reflect not only limitations in drug delivery but also target specificity. TR100 was originally identified for its activity against the mammalian tropomyosin isoform Tpm3.1 and does not broadly inhibit all tropomyosin isoforms (Stehn et al. 2013). Thus, differences between mammalian Tpm3.1 and *C. elegans* LEV-11 may limit TR100 activity in worms, and the absence of a phenotype following TR100 treatment should not be interpreted as evidence that tropomyosin function is dispensable for actin maintenance or longevity. Similarly, although phalloidin binds filamentous actin with high affinity, it produced little effect on lifespan despite inducing only modest changes in actin morphology. Structural differences between phalloidin and the previously characterized actin stabilizer jasplakinolide, which extends lifespan at substantially lower concentrations (Averbukh, et al. 2026), may contribute to differences in cellular uptake or target accessibility.

An important conclusion of this study is the recognition that pharmacological approaches, while attractive because of their translational potential, may offer limited advantages in *C. elegans*. Small molecules remain highly valuable in systems where genetic manipulation is impractical or unavailable, and they more closely resemble therapeutic strategies used in mammalian studies. However, in *C. elegans*, the extensive genetic toolkit, low cost of RNAi, and robust phenotypes produced by genetic perturbation make RNAi-based approaches substantially more practical for studying actin biology. In contrast, many of the compounds tested here were expensive, required relatively high concentrations, and were subject to uncertainties in stability and delivery during prolonged aging experiments. Together, these considerations suggest that pharmacological perturbation may be better suited for validation of specific pathways or studies in other model systems rather than as a primary strategy for investigating actin function during aging in *C. elegans*.

Despite these limitations, this work provides an important framework for evaluating pharmacological modulation of the cytoskeleton during aging. Because actin is essential for virtually every aspect of cell biology, direct targeting of actin itself has often been limited by toxicity (Anand et al. 2023). Consequently, increasing attention has shifted toward selectively targeting actin regulatory proteins to modulate cytoskeletal dynamics while minimizing widespread cellular dysfunction. Our findings demonstrate that pharmacological compounds targeting distinct actin regulatory pathways differ substantially in their ability to produce detectable effects on longevity and cytoskeletal organization in *C. elegans*. Importantly, the failure of several compounds to reproduce established genetic phenotypes emphasizes the need to verify target engagement before interpreting negative pharmacological results as evidence that a pathway is dispensable for aging.

## Supplementary Information

Below is the link to the electronic supplementary material.

Supplementary file1 (XLSX 19 KB)

Supplementary file2 (DOCX 84 KB)

## Data Availability

All data required to evaluate the conclusions in this manuscript are available within the manuscript and Supplementary Materials. All strains synthesized in this manuscript are derivatives of N2 and available on CGC.
