# Supplementary material for: Evaluating pharmacological targeting of actin regulatory pathways during aging in C. elegans
Source: Biogerontology. 2026 Sep 2;27(5):156. doi: 10.1007/s10522-026-10503-3 (PMC13538196; doi:10.1007/s10522-026-10503-3)
Supplement: Supplementary file 1 [file 10522_2026_10503_MOESM2_ESM.docx]

**Fig. S1. 10 µM CK666 reduces lifespan in *C. elegans*.** Lifespan curves of animals exposed from L1 to 10 µM CK666 (red) compared to DMSO (blue). **** = p < 0.0001 using Log-Rank Testing.
